# Spexin as a Novel Biomarker for Predicting Disease Severity and Mortality in Acute Pancreatitis

**DOI:** 10.3390/ijms27041845

**Published:** 2026-02-14

**Authors:** Betül Çiğdem Yortanlı, Ümmügülsüm Can, Tevhide Şahin, Mehmet Yortanlı, Oğuzhan Aksu

**Affiliations:** 1 Department of Internal Medicine, University of Health Sciences, Konya City Hospital, 42060 Konya, Turkey; 2 Department of Biochemistry, University of Health Sciences, Konya City Hospital, 42060 Konya, Turkey; ummugulsum.can@saglik.gov.tr; 3 Division of Gastroenterology, Department of Internal Medicine, University of Health Sciences, Konya City Hospital, 42060 Konya, Turkey; tevhide.sahin@saglik.gov.tr; 4 Department of Emergency Medicine, Konya Numune Hospital, 42020 Konya, Turkey; mehmet.yortanli@saglik.gov.tr; 5 Division of Endocrinology and Metabolism, Department of Internal Medicine, University of Health Sciences, Konya City Hospital, 42060 Konya, Turkey; oguzhan.aksu@saglik.gov.tr

**Keywords:** acute pancreatitis, severe acute pancreatitis, spexin, biomarkers, mortality

## Abstract

This study aimed to investigate the diagnostic and prognostic value of serum spexin levels in patients with acute pancreatitis (AP) and to assess their association with disease severity and mortality. A total of 34 AP patients and 34 healthy individuals were included in the study. AP patients were classified into three groups: mild, moderately severe, and severe (SAP). Serum spexin levels were measured using ELISA, and biochemical parameters, inflammatory indices, and clinical scores were compared. Univariate logistic regression analysis was performed to evaluate factors associated with AP severity and mortality, and ROC analysis was used to assess the diagnostic performance. The median spexin level in AP patients was significantly higher than in healthy controls (*p* < 0.001). Spexin levels increased markedly in SAP patients and were highest in non-survivors (*p* < 0.001). In univariate analysis, higher spexin levels were associated with SAP (OR: 4.116, 95% CI: 1.348–12.572; *p* = 0.013) and 28-day mortality (OR: 3.132, 95% CI: 1.316–7.453; *p* = 0.010). ROC analyses suggested favorable diagnostic performance of spexin; however, given the exploratory design and small sample size, the findings should be interpreted with caution. Spexin may serve as a complementary early-phase biomarker for risk stratification.

## 1. Introduction

Acute pancreatitis (AP) is one of the most common gastrointestinal diseases leading to hospital admissions worldwide, and its incidence is steadily increasing. The disease course varies widely, ranging from mild clinical presentations to multiple organ failure and mortality. Although the diagnosis of AP is based on clinical findings, biochemical tests, and imaging modalities, there remains a lack of reliable early-phase biomarkers capable of accurately predicting disease severity and prognosis at the time of presentation [1,2].

Recent evidence and contemporary reviews also emphasize that currently available inflammatory and pancreatic biomarkers—such as C-reactive protein (CRP), procalcitonin, IL-6, IL-8, TAP, and endothelial injury markers—remain limited in their early predictive performance, underscoring the ongoing need for novel early-phase biomarkers in AP [3].

Spexin is a recently discovered 14-amino acid neuropeptide associated with energy metabolism, appetite regulation, inflammation, and endocrine functions [4,5]. Previous studies have primarily focused on its role in chronic metabolic conditions, including obesity and diabetes, where altered circulating spexin levels have been associated with long-term metabolic dysregulation and energy homeostasis [6]. Experimental and cellular studies have further demonstrated that spexin is expressed in pancreatic tissue and interacts with pancreatic endocrine cells, influencing insulin secretion and glucose-related metabolic responses [7,8]. Given its involvement in inflammatory and oxidative stress-related pathways, spexin has been suggested as a molecule associated with inflammatory activity, primarily in the context of metabolic stress-related conditions [9].

This study aimed to evaluate the diagnostic and prognostic value of serum spexin levels in patients with AP and to examine their association with disease severity. We hypothesized that circulating spexin levels are associated with disease severity in acute pancreatitis.

## 2. Results

A total of 34 AP patients and 34 healthy controls were included in the study. AP patients had a significantly higher mean age than controls (*p* < 0.001). There was no significant difference in gender distribution between groups. Patients were classified into three severity groups, with no differences in age and gender distribution among them. Gallstone-induced AP was the most common etiology, with no significant difference between groups. ICU admission and 28-day mortality occurred only in the SAP group (Table 1 and Table 2).

**Figure 1:**
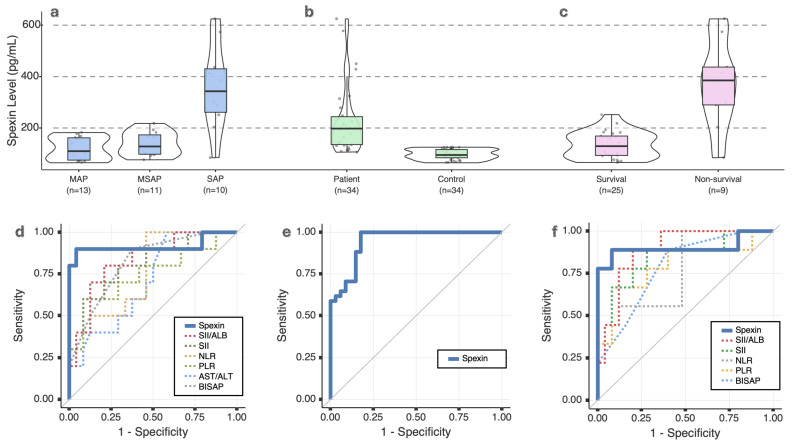
Distribution of serum spexin levels and exploratory ROC analyses in acute pancreatitis. (**a**) Violin and box plots showing serum spexin levels across acute pancreatitis severity groups: mild acute pancreatitis (MAP, *n* = 13), moderately severe acute pancreatitis (MSAP, *n* = 11), and severe acute pancreatitis (SAP, *n* = 10). (**b**) Comparison of serum spexin levels between patients with acute pancreatitis (*n* = 34) and healthy controls (*n* = 34). (**c**) Distribution of serum spexin levels in survivors (*n* = 25) and non-survivors (*n* = 9). (**d**) Receiver operating characteristic (ROC) curves comparing the performance of spexin with BISAP score and inflammatory markers for predicting severe acute pancreatitis. (**e**) ROC curve evaluating the performance of serum spexin levels for the diagnosis of acute pancreatitis. (**f**) ROC curves assessing the prognostic performance of spexin and selected biomarkers for mortality prediction. All ROC analyses were performed for exploratory purposes only. Due to the limited sample size and the absence of an external validation cohort, the diagnostic performance estimates should be interpreted cautiously.

Median serum spexin levels in AP patients were 163.3 pg/mL (94.9–226.5), significantly higher than in controls (*p* < 0.001). SAP patients had the highest median spexin levels at 342.7 pg/mL (239.6–471.5) (*p* < 0.001) (Table 1 and Table 2, Figure 1).

SAP patients had significantly lower albumin and calcium levels. Inflammatory markers, including BISAP, NLR, PLR, SII, SII/ALB, and AST/ALT ratio, were significantly higher in the SAP group (*p* < 0.05) (Table 1).

28-day mortality cases had a median spexin level of 384.8 pg/mL (246.7–505.8), significantly higher than survivors (*p* < 0.001). Other significant predictors of mortality included BISAP, NLR, PLR, SII, and SII/ALB (*p* < 0.05) (Table 3, Figure 1).

According to the univariate logistic regression analysis, spexin levels were found to be significantly associated with both SAP development (OR: 4.116, 95% CI: 1.348–12.572, *p* = 0.013) and 28-day mortality (OR: 3.132, 95% CI: 1.316–7.453, *p* = 0.010) (Table 4). In age-adjusted logistic regression analysis, serum spexin levels remained significantly associated with the presence of AP after adjustment for age (OR: 0.881, 95% CI: 0.804–0.965; *p* = 0.007).

ROC analysis demonstrated that spexin levels have high diagnostic performance for SAP (AUC: 0.917, 95% CI: 0.770–1.000, *p* < 0.001). The optimal cutoff value for spexin was determined as >194 pg/mL, with a sensitivity of 90% and specificity of 95.8%. Spexin also showed strong prognostic value in predicting mortality (AUC: 0.902, 95% CI: 0.736–1.000, *p* < 0.001). The optimal cutoff for mortality prediction was >198 pg/mL, yielding a sensitivity of 88.9% and specificity of 92.0%. Additionally, spexin demonstrated a high diagnostic performance for AP (AUC: 0.946, 95% CI: 0.896–0.995, *p* < 0.001), with an optimal cutoff of >85.5 pg/mL, corresponding to a sensitivity of 82.4%, specificity of 91.2%, a PPV of 90.3%, and a NPV of 83.8% (Table 5, Figure 1).

## 3. Discussion

In this preliminary, hypothesis-generating study with a limited sample size, serum spexin levels were evaluated in patients with AP. In our small cohort, higher spexin levels were observed in patients with severe disease, and spexin showed a promising AUC in ROC analyses, suggesting a potential association with disease severity. Our findings suggest that serum spexin levels are associated with the presence and severity of AP; however, these observations should be interpreted within the context of the study’s exploratory design. Specifically, the significantly higher spexin levels observed in the SAP group suggest that spexin may be associated with disease severity, rather than indicating definitive predictive performance. However, these findings should be interpreted cautiously due to the limited number of severe cases and mortality events, which may contribute to uncertainty in effect size estimates. Because no external validation cohort was available, the present findings should be regarded as preliminary and require confirmation in independent populations.

However, our study has several limitations. Due to the limited number of severe cases and deaths, multivariate logistic regression was not performed to avoid overfitting, and the results should therefore be interpreted as exploratory. In particular, the small number of SAP cases and mortality events resulted in wide confidence intervals in logistic regression analyses, reflecting substantial statistical uncertainty. In addition, the relatively small sample size, particularly with respect to subgroup comparisons and mortality analyses, limits the generalizability of the findings and necessitates validation in larger cohorts. Another important limitation of our study is that the healthy control group was significantly younger than the AP patients. Because older, comorbidity-free volunteers are difficult to recruit, age-matching could not be performed, and therefore the potential confounding effect of age and metabolic status on spexin levels cannot be fully excluded. Given the established associations between spexin and metabolic regulation, age-related metabolic differences between patients and controls may have contributed to variability in circulating spexin levels and should be considered when interpreting the results. Importantly, the association between serum spexin levels and AP remained significant after adjustment for age, suggesting that the observed relationship was not solely attributable to age-related metabolic differences between patients and controls. In addition, multiple univariate comparisons were performed without correction for multiplicity, which may increase the risk of type I error; therefore, the findings should be interpreted with caution. Moreover, the diagnostic performance estimated by ROC analyses may be overestimated due to the limited sample size and the absence of external validation; therefore, the reported AUC values should be interpreted as preliminary. Furthermore, the long-term prognostic effects of spexin levels were not evaluated, and prospective follow-up studies are warranted to clarify how spexin concentrations may change over the course of the disease. Finally, the potential additive diagnostic value of combining spexin with other biomarkers was not assessed, and future studies are required to explore whether such combinations may improve clinical risk stratification.

Spexin has been previously associated with inflammation, energy balance, and metabolic processes, and is considered a multifunctional neuroendocrine peptide involved in systemic homeostasis. Experimental and clinical studies have primarily described altered spexin levels in chronic metabolic and inflammatory conditions, which are characterized by long-term adaptive regulatory changes [10,11,12]. In contrast, AP represents a sudden inflammatory insult accompanied by abrupt metabolic and systemic stress. Given that spexin is expressed in pancreatic tissue and is responsive to dynamic metabolic signals, its circulating levels may exhibit a distinct pattern during acute inflammatory states compared with chronic metabolic disorders [13,14]. This difference suggests that spexin behavior in acute pancreatic inflammation may reflect transient neuroendocrine and metabolic stress responses rather than the sustained regulatory alterations observed in chronic conditions [4,15]. The elevated spexin levels in SAP patients observed in our study are consistent with a possible association between spexin and the systemic inflammatory response observed in SAP.

Although the exact pathophysiological mechanism underlying increased spexin levels in AP is not fully elucidated, the following discussion is intended to provide a contextual interpretation of the associations observed in our study rather than mechanistic conclusions. Spexin is expressed in pancreatic islet cells and has been shown to modulate β-cell viability and proliferation, as well as insulin secretion in experimental models [7,8]. In our cohort, elevated circulating spexin levels were associated with disease severity, which may reflect pancreatic endocrine stress during acute inflammatory injury. Previous studies have also suggested anti-inflammatory and metabolic regulatory roles of spexin [9,11,13], which may provide a biological framework to interpret these correlations. However, these mechanisms were not directly assessed in the present study and should therefore be regarded as speculative and hypothesis-generating rather than confirmatory.

BISAP scoring is widely used for early risk stratification in AP patients. Arif et al. [16] reported that BISAP is valuable in early SAP prediction, but its sensitivity is limited. A meta-analysis by Gao et al. [17] showed that while BISAP has high specificity, it does not achieve optimal sensitivity in predicting mortality and SAP. Similarly, a systematic review by Chandra et al. [18] emphasized that while BISAP is a reliable prognostic tool in different patient populations, it has some limitations when used alone. In our study, elevated spexin levels in SAP patients were observed, suggesting a potential complementary role alongside BISAP in early-phase risk assessment, within the limitations of the present exploratory study. Accordingly, spexin is proposed as a complementary biomarker to existing clinical scores and laboratory parameters, rather than a replacement for established prognostic tools.

From a clinical perspective, serum spexin measurement may be considered as an adjunctive biomarker obtained during the early evaluation of patients presenting with acute pancreatitis. Within this exploratory framework, spexin could potentially be integrated alongside routinely available laboratory parameters and clinical scoring systems to support early risk stratification, rather than replacing established diagnostic or prognostic tools.

In recent years, inflammatory markers such as NLR, PLR, and SII have been evaluated as predictors of AP severity [19,20,21]. However, studies have reported inconsistent sensitivity and specificity levels across different patient populations [22,23]. Moreover, the AST/ALT ratio (De Ritis ratio) has been proposed as a marker of systemic inflammation and organ damage. Ak et al. [24] demonstrated that an elevated AST/ALT ratio is a significant predictor of AP severity. In our study, spexin levels demonstrated measurable diagnostic performance alongside routinely used inflammatory markers, suggesting potential complementary value. These observations indicate that spexin may reflect multiple components of the systemic inflammatory response in AP, although further studies are required to clarify its role and clinical relevance.

Several routinely used inflammatory and pancreatic biomarkers have been evaluated for early prediction of AP severity [3], and their diagnostic performance provides an essential context for interpreting the potential role of spexin. Reviews published in recent years continue to emphasize the limitations of currently available prognostic scoring systems and biomarkers, particularly their restricted sensitivity in the early phase of disease and their variable performance across different patient populations [25,26,27]. In this context, the revised IAP/APA 2025 guidelines emphasize the use of early clinical and inflammatory markers, including CRP and IL-6, for the prediction of SAP within the first 48 h of presentation [3]. CRP is one of the most widely used biomarkers; however, meta-analytic evidence indicates that its diagnostic accuracy is limited during the first 72 h, with performance improving only after levels peak later in the disease course [28,29]. Procalcitonin, another commonly used marker, shows moderate diagnostic accuracy in the early phase, but its clinical utility is restricted by cost and limited assay availability [29,30]. Pro-inflammatory cytokines such as IL-6 and IL-8 rise rapidly during the early inflammatory response and are strongly associated with SAP and organ failure, although their routine clinical use is constrained by short half-life, laboratory complexity, and lack of standardization [31,32]. Trypsinogen activation peptide (TAP), despite its mechanistic relevance, has demonstrated inconsistent performance across clinical studies, with reports of wide inter-individual variability and frequent measurements below the detection threshold in early samples [33]. Angiopoietin-2, a marker of endothelial injury, has shown high diagnostic accuracy for predicting organ failure, but its optimal measurement window generally occurs later in the disease course, limiting its immediate applicability at presentation [34,35]. Collectively, these findings indicate that although conventional biomarkers provide important pathophysiological insight, none offers a consistently reliable, rapid, and widely accessible early diagnostic signal. Within the limitations of the present exploratory study, spexin should be considered a potential complementary early-phase biomarker rather than a superior alternative to established markers. Further studies are needed to determine whether spexin provides incremental prognostic value beyond existing biomarkers.

Ni et al. [36] reported that low albumin and prealbumin levels in SAP patients were associated with poor prognosis and could be used as therapeutic targets. Liu et al. [23] found that the SII/ALB ratio was linked to mortality in SAP patients. These findings were interpreted in the context of existing literature and the potential biological role of spexin in acute inflammatory states.

Overall, the present findings suggest a potential association between serum spexin levels and disease severity in acute pancreatitis. Within the limitations of this exploratory study, spexin should be considered a potential complementary early-phase biomarker rather than a definitive prognostic tool. However, these observations should be interpreted cautiously and require validation in larger, multicenter cohorts before any clinical implications can be inferred.

## 4. Materials and Methods

### 4.1. Study Design and Participants

This prospective, single-center observational study was conducted in the internal medicine ward and intensive care unit (ICU) of a tertiary care hospital. Patients diagnosed with AP and hospitalized between 1 October 2024, and 31 December 2024, were included. Exclusion criteria were patients under 18 years old, those with autoimmune diseases, cardiopulmonary disease, end-stage renal failure, active tuberculosis, cirrhosis, cancer, chronic pancreatitis, pregnancy, or those transferred to another hospital during follow-up. Ethical approval was granted by the KTO Karatay University Faculty of Medicine Ethics Committee on 26 September 2024 (decision number: 2024/007), and the study was conducted in accordance with the Declaration of Helsinki. Informed consent was obtained from all individual participants included in the study.

### 4.2. Data Collection

Demographic information, etiological factors, laboratory parameters, and clinical outcomes of the patients were recorded. AP severity was classified into mild AP (MAP), moderately severe AP (MSAP), and severe AP (SAP). The bedside index for severity in acute pancreatitis (BISAP), neutrophil-to-lymphocyte ratio (NLR), platelet-to-lymphocyte ratio (PLR), and systemic immune inflammation index (SII) were calculated. Healthy individuals without known diseases or signs of infection were included as controls.

Serum spexin levels were measured using a commercial ELISA kit (ELK Biotechnology, Wuhan, China; Catalog No: ELK8816). According to the manufacturer’s instructions, the assay sensitivity was 12.2 pg/mL, with a detection range of 31.25–2000 pg/mL. The intra-assay CV was <8%, and the inter-assay CV was <10%. Venous blood samples were collected within the first 24 h of hospital admission, before any treatment was initiated. All samples were obtained during the early phase of acute pancreatitis, corresponding to the initial presentation following symptom onset. All samples were centrifuged within 30 min, and serum aliquots were immediately stored at −80 °C until analysis. Hemolyzed or lipemic samples were excluded. Serial measurements were not performed, and all analyses were based on baseline samples, as the primary objective of the study was to evaluate the early-phase association between serum spexin levels and disease severity at initial presentation. Laboratory personnel performing the spexin measurements were blinded to both the clinical severity classification and the study group allocation.

### 4.3. Definitions

Patients were diagnosed with AP and classified according to the 2012 Atlanta Classification [37]. Diagnosis was based on at least two of the following criteria: acute onset of severe epigastric pain often radiating to the back, serum lipase or amylase levels at least three times the upper normal limit, or imaging findings consistent with AP on contrast-enhanced CT, MRI, or abdominal ultrasound. MAP was defined as AP without organ failure or complications, MSAP as AP with transient organ failure resolving within 48 h and/or local or systemic complications, and SAP as AP with persistent organ failure lasting more than 48 h. SII was calculated using the formula (neutrophil × platelet)/lymphocyte. BISAP score was determined based on five variables: blood urea nitrogen (BUN) > 25 mg/dL, altered mental status or Glasgow Coma Scale (GCS) < 15, systemic inflammatory response syndrome (SIRS), age > 60, and pleural effusion on imaging, with each variable scoring 1 point.

### 4.4. Statistical Analysis

Sample size estimation was performed using G*Power software (version 3.1.9.7; Heinrich Heine University Düsseldorf, Düsseldorf, Germany) [38]. The calculation was based on previously published data by Khadir et al. [4], in which a mean difference of approximately 180 pg/mL in circulating spexin levels between two groups was reported, with a standard deviation (SD) of 180 pg/mL. Assuming a two-sided significance level of 0.05 and a statistical power of 98% (1 − β = 0.98), the estimated total sample size required to detect a significant difference using an independent samples *t*-test was 68 participants (34 patients with AP and 34 healthy controls).

All statistical analyses were performed using SPSS (Statistical Package for the Social Sciences; version 27; IBM Corp., Armonk, NY, USA) and Jamovi (version 2.4.11; The Jamovi Project, Sydney, Australia). Normality was assessed using the Kolmogorov–Smirnov and Shapiro–Wilk tests. Normally distributed variables were presented as mean ± SD, while non-normally distributed variables were expressed as median (interquartile range). Categorical variables were reported as frequency (%). Comparisons between two groups were conducted using the independent *t*-test for normally distributed variables and the Mann–Whitney U test for non-normally distributed variables. ANOVA was used for multiple group comparisons with normal distribution, while the Kruskal–Wallis test and post hoc tests were applied for non-normal distributions. For pairwise post hoc comparisons following the Kruskal–Wallis test, Dunn’s test with Bonferroni-adjusted *p*-values was applied. Categorical variables were analyzed using the Chi-square test or Fisher’s exact test. Only univariate logistic regression analyses were performed to identify factors associated with SAP and mortality. To evaluate whether the association between serum spexin levels and AP was independent of age, an age-adjusted binary logistic regression analysis was additionally performed, including spexin levels and age as covariates. Given the exploratory nature of this study and the limited sample size, no formal adjustment for multiple comparisons was applied; *p* values should therefore be interpreted descriptively. Odds ratios (ORs) and 95% confidence intervals (CIs) were calculated. Receiver Operating Characteristic (ROC) curve analysis was conducted to evaluate the diagnostic performance of spexin and other biomarkers, and Area Under the Curve (AUC) values were calculated. The optimal cutoff values, sensitivity, specificity, positive predictive value (PPV), and negative predictive value (NPV) were determined. A *p*-value of <0.05 was considered statistically significant.

## 5. Conclusions

In conclusion, serum spexin levels are associated with disease severity and mortality in patients with AP. These findings suggest that spexin may have potential value as a complementary early-phase biomarker; however, given the exploratory nature, limited sample size, and single-center design of the present study, its clinical applicability should be interpreted cautiously. Larger, multicenter studies are required to confirm these observations and to determine whether spexin provides incremental value alongside established biomarkers.

## Figures and Tables

**Table 1:** Demographic and clinical characteristics of acute pancreatitis patients.

Parameters	Total (*n* = 34)	MAP (*n* = 13)	MSAP (*n* = 11)	SAP (*n* = 10)	*p*-Value
Age, years	61 ± 18	53 ± 17	60 ± 20	71 ± 13	0.067
Gender, *n* (%)					
Male	9 (26.5)	2 (22.2)	5 (56.6)	2 (22.2)	0.215
Female	25 (73.5)	11 (44)	6 (24)	8 (32)	
Etiology, *n* (%)					
Gallstones	19 (55.9)	7 (36.8)	5 (26.3)	7 (36.8)	0.463
Alcohol	4 (11.8)	0 (0.0)	2 (50.0)	2 (50.0)	
Hypertriglyceridemia	2 (5.9)	1 (50.0)	1 (50.0)	0 (0.0)	
Other	9 (26.5)	5 (55.6)	3 (33.3)	1 (11.1)	
BISAP score	2 (1–2)	1 (0–2)	1 (1–3)	3 (2–3)	0.003
ICU length of stay, day	10 (5–24)	-	-	10 (5–24)	-
Length of hospital stay, day	8 (5–12)	6 (5–10)	8 (5–12)	13 (8–24)	0.104
28-day mortality, *n* (%)	9 (26.5)	0 (0.0)	0 (0.0)	9 (100.0)	<0.001
Laboratory test					
WBC, 10^3^/µL	11.1 ± 3.6	11.8 ± 4.6	10.2 ± 3.2	11.2 ± 2.5	0.559
Neutrophil, 10^3^/µL	7.8 (6.1–9.8)	7.7 (5.5–9.4)	7.3 (5.9–8.4)	9.1 (7.4–16.8)	0.075
Lymphocyte, 10^3^/µL	1.7 ± 0.4	1.9 ± 0.3	1.6 ± 0.4	1.5 ± 0.4	0.010
Platelet, 10^3^/µL	223 (186–290)	222 (195–267)	210 (170–311)	280 (209–330)	0.232
Hemoglobin, g/dL	13.6 ± 1.8	14.0 ± 1.8	13.0 ± 2.0	13.9 ± 1.5	0.363
Creatinine, mg/dL	0.9 (0.7–1.3)	0.7 (0.7–1.0)	0.9 (0.6–1.5)	1.1 (0.9–1.5)	0.128
Glucose, mg/dL	90 ± 13	93 ± 10	87 ± 15	91 ± 13	0.593
ALB, g/dL	3.4 (3.1–3.7)	3.6 (3.4–3.8)	3.2 (2.7–3.6)	3.4 (2.9–3.9)	0.046
AST, U/L	124 (47–427)	69 (28–153)	121 (47–195)	243 (148–293)	0.025
ALT, U/L	134 (37–218)	75 (24–184)	140 (24–173)	176 (68–258)	0.354
Amylase, U/L	1458 (1077–2293)	1279 (825–1916)	2033 (1092–2360)	1795 (1109–2674)	0.201
Lipase, U/L	1802 (909–3016)	1509 (271–5148)	1350 (902–2658)	2364 (1213–3307)	0.417
Triglyceride, mg/dL	140 (102–176)	128 (94–167)	135 (110–162)	166 (99–219)	0.624
Calcium, mg/dL	8.4 (7.7–8.7)	8.3 (7.8–8.7)	7.4 (7.2–7.9)	7.3 (7.1–8.0)	0.006
CRP, mg/dL	124 (51–162)	119 (46–171)	123 (46–160)	153 (108–172)	0.663
AST/ALT ratio	1.2 (0.9–1.7)	0.9 (0.8–1.3)	1.4 (0.9–2.9)	1.4 (1.0–2.7)	0.041
NLR	4.6 (3.6–6.9)	3.9 (2.8–5.7)	4.6 (3.4–6.9)	6.4 (4.5–9.7)	0.046
PLR	127.4 (108.8–185.0)	121.7 (106.2–129.8)	148.0 (100.0–196.3)	185.0 (122.6–268.2)	0.044
SII	1033 (834–1594)	962 (572–1134)	942 (631–1433)	1733 (1104–2619)	0.015
SII/ALB ratio	31.7 (21.9–51.7)	26.7 (15.9–30.9)	32.3 (25.1–53.5)	44.3 (35.0–83.7)	0.003
Spexin, pg/mL	163.3 (94.9–226.5)	109.8 (72.4–165.2)	128.1 (95.4–182.7)	342.7 (239.6–471.5)	<0.001

MAP: mild acute pancreatitis; MSAP: moderately acute pancreatitis; SAP: severe acute pancreatitis; BISAP: bedside index for severity in acute pancreatitis; ICU: intensive care unit; WBC: white blood cell; ALB: albumin; AST: aspartate aminotransferase; ALT: alanine aminotransferase; CRP: C-reactive protein; NLR: neutrophil/lymphocyte ratio; PLR: platelet/lymphocyte ratio; SII: systemic immune-inflammation index. Post hoc analysis of statistically significant groups; BISAP: MAP~MSAP *p* = 0.079, MAP~SAP *p* = 0.001, MSAP~SAP *p* = 0.051; Lymphocyte: MAP~MSAP *p* = 0.026, MAP~SAP *p* = 0.478, MSAP~SAP *p* = 0.041; ALB: MAP~MSAP *p* = 0.013, MAP~SAP *p* = 0.171, MSAP~SAP *p* = 0.358; AST: MAP~MSAP *p* = 0.354, MAP~SAP *p* = 0.013, MSAP~SAP *p* = 0.041; Calcium: MAP~MSAP *p* = 0.008, MAP~SAP *p* = 0.009, MSAP~SAP *p* = 0.571; AST/ALT ratio: MAP~MSAP *p* = 0.118, MAP~SAP *p* = 0.012, MSAP~SAP *p* = 0.481; NLR: MAP~MSAP *p* = 0.434, MAP~SAP *p* = 0.013, MSAP~SAP *p* = 0.121; PLR: MAP~MSAP *p* = 0.235, MAP~SAP *p* = 0.013, MSAP~SAP *p* = 0.205; SII: MAP~MSAP *p* = 0.469, MAP~SAP *p* = 0.005, MSAP~SAP *p* = 0.041; SII/ALB ratio: MAP~MSAP *p* = 0.099, MAP~SAP *p* < 0.001, MSAP~SAP *p* = 0.067; Spexin: MAP~MSAP *p* = 0.259, MAP~SAP *p* < 0.001, MSAP~SAP *p* = 0.002.

**Table 2:** Spexin levels and demographic distribution of acute pancreatitis patients and control group.

Parameters	Total (*n* = 68)	Patient (*n* = 34)	Control (*n* = 34)	*p*-Value
Age, years	48 ± 19	61 ± 18	36 ± 9	<0.001
Gender, *n* (%)				
Male	20 (29.4)	9 (45.0)	11 (55.0)	0.595
Female	48 (70.6)	25 (52.1)	23 (47.9)	
Spexin, pg/mL	82.0 (52.5–163.9)	163.3 (94.9–226.5)	53.1 (39.5–77.0)	<0.001

**Table 3:** Clinical and laboratory characteristics of surviving and non-surviving patients with acute pancreatitis.

Parameters	Survival (*n* = 25)	Non-Survival (*n* = 9)	*p*-Value
Age, years	57 ± 18	72 ± 14	0.030
Gender, *n* (%)			
Male	7 (77.8)	2 (22.2)	0.736
Female	18 (72.0)	7 (28.0)	
Etiology, *n* (%)			
Gallstones	13 (68.4)	6 (31.6)	0.361
Alcohol	2 (50.0)	2 (50.0)	
Hypertriglyceridemia	2 (100.0)	0 (0.0)	
Other	8 (88.9)	1 (11.1)	
BISAP score	1 (1–2)	2 (2–4)	0.008
ICU length of stay, day	-	10 (7–25)	-
Length of hospital stay, day	7 (5–11)	14 (7–25)	0.104
Laboratory test			
WBC, 10^3^/µL	11.1 ± 3.9	11.1 ± 2.7	0.987
Neutrophil, 10^3^/µL	7.6 (5.8–8.7)	9.1 (7.2–16.9)	0.033
Lymphocyte, 10^3^/µL	1.8 ± 0.4	1.5 ± 0.4	0.061
Platelet, 10^3^/µL	222 (185–278)	296 (234–338)	0.023
Hemoglobin, g/dL	13.5 ± 1.9	14.0 ± 1.5	0.482
Creatinine, mg/dL	0.9 (0.7–1.1)	1.1 (0.9–1.6)	0.054
Glucose, mg/dL	90 ± 12	91 ± 14	0.903
ALB, mg/dL	35 (32–37)	33 (28–39)	0.434
AST, U/L	104 (36–194)	241 (139–263)	0.040
ALT, U/L	127 (25–191)	141 (63–243)	0.329
Amylase, U/L	1305 (1054–2258)	1947 (1170–2696)	0.205
Lipase, U/L	1509 (658–2860)	2032 (1134–3555)	0.301
Triglyceride, mg/dL	135 (102–162)	189 (92–220)	0.329
Calcium, mg/dL	7.9 (7.4–8.5)	7.3 (7.1–8.2)	0.147
CRP, mg/dL	120 (45–162)	154 (125–183)	0.114
AST/ALT ratio	1.1 (0.8–1.6)	1.2 (1.0–2.9)	0.138
NLR	4.1 (3.3–6.5)	6.9 (4.4–10.0)	0.022
PLR	123.3 (106.2–143.2)	185.0 (130.9–269.3)	0.011
SII	962 (617–1227)	1783 (1236–2794)	0.003
SII/ALB ratio	28.5 (20.8–34.9)	49.2 (37.8–87.6)	<0.001
Spexin, pg/mL	128.1 (92.8–172.9)	384.8 (246.7–505.8)	<0.001

ICU: intensive care unit; WBC: white blood cell; ALB: albumin; AST: aspartate aminotransferase; ALT: alanine aminotransferase; CRP: C-reactive protein; NLR: neutrophil/lymphocyte ratio; PLR: platelet/lymphocyte ratio; SII: systemic immune-inflammation index.

**Table 4:** Univariate logistic regression analysis for predicting severe acute pancreatitis and mortality.

Variables	Univariate Logistic Regression	
	OR (95% Cl)	*p*-Value
SAP		
Spexin	4.116 (1.348–12.572)	0.013
SII/ALB ratio	1.049 (1.007–1.092)	0.022
SII	1.002 (1.000–1.003)	0.020
NLR	1.413 (1.013–1.972)	0.042
PLR	1.021 (1.003–1.038)	0.019
AST/ALT ratio	2.507 (0.950–6.612)	0.063
BISAP score	2.018 (1.207–3.376)	0.007
28-day mortality		
Spexin	3.132 (1.316–7.453)	0.010
SII/ALB ratio	1.056 (1.011–1.104)	0.014
SII	1.002 (1.000–1.003)	0.015
NLR	1.421 (1.013–1.994)	0.042
PLR	1.024 (1.005–1.044)	0.013
BISAP score	1.808 (1.126–2.904)	0.014

ALB: albumin; AST: aspartate aminotransferase; ALT: alanine aminotransferase; BISAP: bedside index for severity in acute pancreatitis; NLR: neutrophil/lymphocyte ratio; PLR: platelet/lymphocyte ratio; SAP: severe acute pancreatitis; SII: systemic immune-inflammation index.

**Table 5:** Predictive performance of spexin and other biomarkers for severe acute pancreatitis and mortality.

	AUC (95% CI)	*p*-Value	Cut-Off	Sensitivity	Specificity	PPV	NPV
SAP							
Spexin	0.917 (0.770–1.000)	<0.001	>194	90.0	95.8	90.0	95.8
SII/ALB ratio	0.833 (0.686–0.980)	0.002	>36	80.0	79.2	61.5	90.5
SII	0.808 (0.645–0.971)	0.005	>1090	80.0	70.8	53.3	89.5
NLR	0.758 (0.590–0.926)	0.019	>4.4	80.0	54.2	42.1	86.7
PLR	0.742 (0.543–0.941)	0.028	>170	60.0	87.5	66.7	84.0
AST/ALT ratio	0.710 (0.530–0.890)	0.056	>1.02	80.0	50.0	40.0	85.7
BISAP score	0.821 (0.672–0.970)	0.004	>2	90.0	62.5	50.0	93.8
28-day mortality							
Spexin	0.902 (0.736–1.000)	<0.001	>198	88.9	92.0	80.0	95.8
SII/ALB ratio	0.889 (0.778–1.000)	0.001	>35	80.0	75.0	57.1	90.0
SII	0.840 (0.678–1.000)	0.003	>1093	80.0	70.8	53.3	89.5
NLR	0.760 (0.582–0.938)	0.022	>4.2	77.8	48.0	65.0	87.7
PLR	0.791 (0.598–0.984)	0.011	>171	66.7	88.0	66.7	88.0
BISAP score	0.789 (0.625–0.953)	0.011	>2	88.9	60.0	44.4	93.8

SAP: severe acute pancreatitis; AUC: area under the curve; PPV: positive predictive value; NPV: negative predictive value; PLR: platelet/lymphocyte ratio; ALB: albumin; AST: aspartate aminotransferase; ALT: alanine aminotransferase; SII: systemic immune-inflammation index; NLR: neutrophil/lymphocyte ratio. The cut-off value for spexin is expressed in pg/mL.

## Data Availability

The data presented in this study are available on reasonable request from the corresponding author. The data are not publicly available due to ethical and privacy restrictions.

## Abbreviations

The following abbreviations are used in this manuscript:

AP	Acute pancreatitis
AST/ALT	Aspartate aminotransferase-to-alanine aminotransferase ratio
AUC	Area under the curve
BISAP	Bedside Index for Severity in Acute Pancreatitis
CRP	C-reactive protein
ELISA	Enzyme-linked immunosorbent assay
ICU	Intensive care unit
MAP	Mild acute pancreatitis
MSAP	Moderately severe acute pancreatitis
NLR	Neutrophil-to-lymphocyte ratio
NPV	Negative predictive value
PLR	Platelet-to-lymphocyte ratio
PPV	Positive predictive value
ROC	Receiver operating characteristic
SAP	Severe acute pancreatitis
SII	Systemic immune–inflammation index
SII/ALB	Systemic immune–inflammation index-to-albumin ratio
